# A Virtual Machine Consolidation Algorithm Based on Dynamic Load Mean and Multi-Objective Optimization in Cloud Computing

**DOI:** 10.3390/s22239154

**Published:** 2022-11-25

**Authors:** Pingping Li, Jiuxin Cao

**Affiliations:** School of Cyber Science and Engineering, Southeast University, Nanjing 211189, China

**Keywords:** cloud computing, VM consolidation, energy saving, multiple objective optimization, load mean

## Abstract

High energy consumption and low resource utilization have become increasingly prominent problems in cloud data centers. Virtual machine (VM) consolidation is the key technology to solve the problems. However, excessive VM consolidation may lead to service level agreement violations (SLAv). Most studies have focused on optimizing energy consumption and ignored other factors. An effective VM consolidation should comprehensively consider multiple factors, including the quality of service (QoS), energy consumption, resource utilization, migration overhead and network communication overhead, which is a multi-objective optimization problem. To solve the problems above, we propose a VM consolidation approach based on dynamic load mean and multi-objective optimization (DLMM-VMC), which aims to minimize power consumption, resources waste, migration overhead and network communication overhead while ensuring QoS. Fist, based on multi-dimensional resources consideration, the host load status is objectively evaluated by using the proposed host load detection algorithm based on the dynamic load mean to avoid an excessive VM consolidation. Then, the best solution is obtained based on the proposed multi-objective optimization model and optimized ant colony algorithm, so as to ensure the common interests of cloud service providers and users. Finally, the experimental results show that compared with the existing VM consolidation methods, our proposed algorithm has a significant improvement in the energy consumption, QoS, resources waste, SLAv, migration and network overhead.

## 1. Introduction

With the rapid development of cloud computing, the scale of data centers becomes larger and larger, and a large number of hosts around the world consume a huge amount of power every day, resulting in high CO_2_ emissions [1]. Studies have shown that the average CPU utilization of physical hosts in cloud data centers is only 15–20%, and the energy consumed by an idle server is 70% of the peak energy [2,3], which not only wastes energy and resources but also greatly increases the operating costs of cloud service providers. In addition, VMs perform data intensive applications by accessing physical networks and communicating with interdependent VMs on different servers, which not only increases the network traffic but also reduces the overall performance of the data center’s network [4]. For users, this seriously hinders the customer’s experience, while for cloud service providers, a QoS may not be guaranteed, leading to SLAv. Therefore, considering the interests of cloud service providers and users, the above issues generate a complex and challenging multi-objective resource management problem in cloud computing.

The cloud computing resources are provided to users in the form of a VM. Therefore, the core problem of resource management in cloud computing is the VMs management, and VM consolidation technology is the main method used to solve this problem. The key to VM consolidation is to dynamically obtain the optimal mapping between VMs and hosts, so as to minimize the energy consumption and resources waste to reduce the operating costs while ensuring a QoS for customers. 

The main process of VM consolidation is to first determine the host load, then decide whether to migrate the VMs according to the host load status, and finally migrating the VMs to a new host. However, a dynamic cloud environment brings more challenges to an effective VM consolidation. First, in a dynamic cloud environment, especially in a cloud data center with thousands of hosts, detecting the host load state effectively and accurately has been an important problem to be solved. Some related studies [3,5,6] determine the host load status based on static thresholds, which lack the awareness of dynamic workloads in the data center. The current resources utilization cannot objectively and truly reflect the real load of hosts, which will lead to an excessive consolidation. The literature [7] proposes a dynamic threshold method, which considers the workload changes in the source host and target host after the VMs migration, but does not consider the resource load balance, which will lead to a large resources waste. In addition, some of the existing methods determine the host load state only based on the CPU resource [3,4,5,6,7,8,9,10], ignoring the impact of memory, network and other resources, which are also key factors affecting the QoS. Some studies [11,12,13,14] consider the impact of multi-dimensional resources, but mostly focus on the optimization of energy consumption and SLAv, ignoring the VM migration overhead and the network communication overhead. For example, during the process of a VMs migration, the performance of the VM will be reduced, and placing two interdependent VMs at a long distance will cause a large communication delay [15].

VM consolidation is a well-known NP-hard problem [16]. Some studies have used heuristic algorithms to solve this question [3,17,18,19]. Heuristic algorithms are widely used in VM consolidation because of their simple implementation and low complexity. However, traditional heuristic algorithms are prone to fall into local optimal solutions. In the existing research, meta-heuristic algorithms are used to solve the VM placement problem [16,20,21,22], such as ant colony optimization (ACO), artificial bee colony, genetic algorithm, etc. These algorithms can effectively avoid local optimal solutions when solving large-scale model problems. The ant colony system (ACS) is a classical method of ACO. It can get a better solution for complex combinatorial problems in an appropriate time. Because of its excellent performance in solving NP-hard and combinatorial optimization problems [23,24], it has attracted more and more attention in solving VM consolidation problems. In this paper, the VM consolidation is considered to be a multi-objective combinatorial optimization problem. Therefore, the ACS is selected as the solution. However, with the increasing size of the data centers, some studies [25,26,27] using the ACS to solve the VM consolidation problem have resulted in too long an execution time due to the increasing search space of the ACS as well. Therefore, it is necessary to further optimize the search performance.

To solve the above problems, this paper proposes a VM consolidation algorithm based on the dynamic load mean and multi-objective optimization in cloud computing, in which the host load status is comprehensively measured based on multi-dimensional resources, and the dynamic characteristics of the system load are considered. Then, the optimized ant colony algorithm is used to obtain the optimal mapping between the VMs and the hosts so as to realize the multiple objectives optimization of the resource utilization, energy consumption, migration and communication overhead. Our main contributions are as follows:

(1) A host load detection method based on the dynamic load mean is proposed which uses multi-dimensional resources to comprehensively measure the host load status and considers the impact of load fluctuations to avoid an excessive consolidation.
(2) A network-aware model is proposed to optimize the network communication overhead of interdependent VMs and the overall network traffic of the data center.
(3) An improved ant colony optimization algorithm is proposed to obtain a better solution and execution efficiency through the optimization of the heuristic factors and execution process.

The rest of the paper is organized as follows. Section 2 describes the related work. Section 3 introduces the relevant models. Section 4 introduces the host load detection method based on the dynamic load mean. Section 5 introduces the DLMM-VMC algorithm in detail. Section 6 gives the experimental results and performance evaluation. Section 7 concludes the paper and discusses the future work.

## 2. Related Work

A VM consolidation mainly solves three problems. That is, how to determine the host load status which mainly involves the host load detection methods, how to select the migration VMs which mainly involves the migration overhead calculation and how to select the placement hosts which mainly involves the best target host selection for the migration of the VMs.

For how to determine the host load status, some studies [3,5,6,28,29] used static thresholds of CPU utilization to determine whether a host was overloaded or underloaded, keeping the CPU utilization of the host between two fixed thresholds. However, in a cloud data center, resource utilization is constantly changing in multiple resource dimensions. In such as dynamic cloud environment, setting static thresholds or using the current utilization of a single resource is not an effective approach, which leads to an excessive consolidation. Therefore, some dynamic threshold algorithms were proposed. Beloglazov et al. [17] proposed an adaptive upper and lower thresholds method that sorted hosts based on the historical CPU data statistical analysis and CPU utilization prediction, which improves the ability to sense the dynamic changes in the host load. However, the load fluctuation was not considered. Chen et al. [30] proposed a host load detection method based on the time sliding window, which recorded the host CPU utilization in a certain time window through a regular sampling. When the host CPU utilization continuously exceeded the predefined threshold, the host was determined to be overloaded. Yadav et al. [31] proposed two adaptive methods based on robust regression to dynamically set the thresholds. Zhou et al. [32] proposed a dynamic adaptive three threshold host load detection method, using a K-Means clustering algorithm to divide the hosts into four types. However, these methods use CPU utilization as the main criterion to determine the host load. Therefore, it is not possible to accurately describe the load status of the hosts with multi-dimensional resource characteristics, which ultimately leads to an unnecessary migration and resources waste.

For how to select the migration of VMs, in [17,33], the authors proposed several migration VM selection strategies, which have been widely used. These strategies include the maximum utilization (MU) strategy, which selects a VM that has the highest CPU usage; the random selection (RS) strategy, which selects a VM randomly based on a uniformly distributed discrete random variable; the maximum correlation (MC) strategy, which selects a VM with the highest correlation with other VMs; and the minimum migration time (MMT) strategy, which selects a VM with the shortest migration time. Li et al. [34] used the similarity between memory contents of VMs to select a migration VM. This method aimed to select a VM with the highest similarity in memory contents from different hosts to reduce the migrated data and time. Masoumzadeh et al. [35] proposed a VM selection strategy based on fuzzy Q-learning, where multiple VM selection technologies were integrated, and the VM selection strategy was selected dynamically based on the fuzzy logic theory according to the current state of a host. Laili et al. [36] proposed a selection mechanism based on the iterative prediction algorithm, which used a reverse selection mechanism to select the most suitable VM from the candidate VM set for each randomly selected host. However, these studies only consider the migration overhead when selecting migration VMs, ignoring the impact on the host load. For overloaded hosts, the overload status should be quickly and accurately eliminated with the minimum migration overhead, while for low loaded hosts, the resources usage should be quickly reduced to shut down the host as soon as possible to reduce the energy consumption.

For how to select the placement hosts, the literatures [3,18] studied how to use the heuristic greedy algorithm to solve the VM placement problem. For instance, with the first fit (FF), first fit decreasing (FFD), best fit (BF) and best fit decreasing (BFD) algorithms [30,31], which aim to reduce the number of running hosts and the VM migration number. However, classical heuristic algorithms are not convenient for VM consolidation, so many subsequent studies have improved these algorithms to make them applicable to VM consolidation. Beloglazov et al. [17] proposed a power-aware best-fit decreasing (PABFD) VM placement algorithm, which selects a host with the least energy consumption increase as a placement host. Li et al. [37] proposed virtual switch aware BFD and FFD algorithms, which comprehensively considered the traffic between VMs and the CPU overhead generated by virtual switches. Moges et al. [38] proposed a modified best fit decreasing (MBFD) algorithm to improve SLAv and active hosts. Zhang et al. [39] further optimized the MBFD algorithm by combining FF and MBFD to achieve a better energy efficiency.

The heuristic greedy algorithm has a low complexity, but it is not suitable for solving large-scale problems and cannot be well applied to large-scale data centers. The meta-heuristic algorithm has significant advantages in solving such problems. Li et al. [13] proposed a QoS-aware and multi-objective dynamic VM consolidation (QMOD) based on improved the genetic algorithm, which optimizes the three objectives of the load balancing, migration overhead and QoS. Li et al. [20] constructed the VM consolidation problem as a multi-objective optimization problem with multi resource constraints and solved the problem based on the artificial bee colony algorithm. They also proposed a VM consolidation method based on differential evolution (DE) [22]. However, in these methods, only the energy consumption and host overload risk are considered. Al-Moalmi et al. [40] proposed a VM placement method based on grey wolf optimization (GWO), which can use the CPU and RAM resources more effectively and reduce the number of active hosts, energy consumption and SLAv. Aryania et al. [26] proposed an energy-aware VM consolidation based on the ACS, which takes the energy consumption caused by VM migration as an important optimization goal. Farahnakian et al. [27] proposed an ACS-based VM consolidation approach that aims to maximize the number of dormant hosts and minimize the number of VM migrations. However, the above VM consolidation algorithms based on ant colony optimization take too long to execute for larger-scale data centers due to the large search space. Xiao et al. [25] proposed an improved ACS to solve the VM consolidation problem; they used the design heuristic factors to select both migration VMs and placement hosts and limited the search space of ants according to the type of host load state, thus reducing the blindness of the ant search and optimizing the execution efficiency. However, they ignore the optimization of selecting migration VMs, and the selection of migration VMs and placement hosts in VM consolidation jointly determines the search performance and execution efficiency of the ACS. Moreover, the above algorithms do not consider resources waste and network overhead. To some extent, the unbalanced resources utilization will greatly increase the resources waste, leading to an increase in the active hosts, thus increasing the energy consumption. In addition, the increase in the network overhead will greatly delay the response time of the application in the VM, which is easy to lead to a QoS degradation. Therefore, VM consolidation should comprehensively consider the interests of both the cloud service providers and the users to ensure that the cloud service providers minimize the operational costs while also ensuring a QoS for the users.

## 3. System Model

### 3.1. Data Center Resource Representation Model

Assume $H=\left\{h_{1},h_{2},\cdots ,h_{j},\cdots ,h_{n}\right\}$ is the collection of a data center’s hosts, where n is the number of hosts, each host has D-dimensional resources and $C_{h_{j}}^{d},d\in \left\{1,2,\cdots ,D\right\}$ represents the capacity of resources d of a host $h_{j}$. $V=\left\{v_{1},v_{2},\cdots ,v_{i},\cdots ,v_{m}\right\}$ is the collection of the data center’s VMs, where m is the number of VMs; similarly, each VM also has D-dimensional resources, and $C_{v_{i}}^{d},d\in \left\{1,2,\cdots ,D\right\}$ represents the capacity of resources d of a VM $v_{i}$.

Further, assume $VM\left(h_{j}\right)$ indicates the VM collection of a host $h_{j}$, $U_{t}^{d}\left(v_{i}\right)$ represents the actual utilization of resource d of a VM $v_{i}$ at time t, and $U_{t}^{d}\left(h_{j}\right)$ is the actual utilization of resource d of a host $h_{j}$ at time t, which can be expressed as Equation (1).

(1) $$U_{t}^{d}\left(h_{j}\right)=\frac{1}{C_{h_{j}}^{d}}\;\sum_{v_{i}\in VM\left(h_{j}\right)}\;U_{t}^{d}\left(v_{i}\right)\times C_{v_{i}}^{d}$$

### 3.2. Energy Consumption Model

The energy consumption of a host in a data center is mainly composed of its related components CPU, memory, hard disk and network, but studies have shown that CPU is the main energy consuming device, and there is a linear relationship between CPU utilization and host energy consumption [3]. Therefore, we can establish the following energy consumption model, as shown below.

(2) $$P_{j}=k\times P_{j}^{max}+\left(1-k\right)\times P_{j}^{max}\times U_{t}^{d}\left(h_{j}\right)$$

where $P_{j}^{max}$ is the energy consumption of a host $h_{j}$ when it is fully loaded, $U_{t}^{d}\left(h_{j}\right)$ is the cpu utilization of a host $h_{j}$ at time t, k is the energy consumption factor and studies show that the energy consumption overhead when the hosts are idle is 70% of that when they are fully loaded, so k is generally set to 0.7 [3]. In addition, through the above analysis, we can see that idle servers generate more energy consumption, and these hosts can be shut down in time to reduce the energy consumption.

Therefore, the total energy consumption in the data center is calculated as follows.

(3) $$P=\sum_{j=1}^{n}P_{j}$$

### 3.3. Resources Waste Model

Each host in the data center may run multiple VMs at the same time, and different VMs may run different applications, so the resources requirements in various dimensions are different. An unreasonable resources allocation will increase the resources waste. Therefore, it is important to ensure that the remaining resources of each dimension on the host are balanced in order to fully utilize the resources and prevent the resources waste. So, we propose a resources waste model, as shown below.

(4) $$W\left(h_{j}\right)=\sum_{\forall d_{x}\in D,\forall d_{y}\in D}\left[\left|\left(1-U_{t}^{d_{x}}\left(h_{j}\right)\right)-\left(1-U_{t}^{d_{y}}\left(h_{j}\right)\right)\right|\right],\;d_{x}\neq d_{y}$$

(5) $$W=\sum_{j=1}^{n}W_{j}$$

where $U_{t}^{d_{x}}\left(h_{j}\right)$ and $U_{t}^{d_{y}}\left(h_{j}\right)$ represent the utilization of resources $d_{x}$ and $d_{y}$ on a host $h_{j}$, respectively. $W\left(h_{j}\right)$ and W denote the resources waste of the host $h_{j}$ and the data center, respectively.

### 3.4. Communication Overhead Model

The more links between VMs which pass through, the greater the network latency, which is one of the most important factors affecting the QoS. Therefore, by optimizing the communication path between VMs, the network latency can be reduced. The total network communication overhead N is calculated based on the network communication overhead between pairs of VMs and the distance to the physical hosts where they are located, as shown in the following equation.

(6) $$W=\sum_{j=1}^{n}W_{j}$$

where $a\left(v_{i},v_{j}\right)$ indicates the network communication overhead between $v_{i}$ and $v_{j}$, $h\left(v_{i}\right)$ and $h\left(v_{j}\right)$ denote the hosts where $v_{i}$ and $v_{j}$ are located, respectively, and $b\left(h\left(v_{i}\right),h\left(v_{j}\right)\right)$ denotes the network communication distance between $h\left(v_{i}\right)$ and $h\left(v_{j}\right)$, which is measured using the number of switches and routes passed by both in the communication process; the larger the value, the greater the network communication overhead.

Placing pairs of VMs with a high network communication overhead on the same or closer hosts can reduce the network communication overhead and greatly reduce the communication latency.

### 3.5. Migration Overhead Model

A VM migration overhead is also a very important optimization objective, because VM migration consumes additional compute resources, and excessive VM migrations can also lead to large workloads and energy consumption. What’s worse, VM migration can degrade the QoS. Therefore, the number of VM migrations should be minimized during VM consolidation.

(7) $$M=\sum_{i=1}^{m}m\left(v_{i}\right),\forall m\left(v_{i}\right)=1$$

where $m\left(v_{i}\right)=1$ indicates that VM $v_{i}$ needs to be migrated and M indicates the total number of migration VMs.

### 3.6. Multi-Objective Optimization

Minimizing the energy consumption, network overhead, migration overhead and resources waste are the multiple optimization objectives to be obtained for VM consolidation in this paper. According to Equations (3) and (5)–(7), we get the following multi-objective optimization model with some constraints.

(8) $$\min F=\vartheta_{1}P+\vartheta_{2}W+\vartheta_{3}N+\vartheta_{4}M$$

Constraints:

(9) $$x_{v_{i}h_{j}}=\{\begin{array}{c} 1,\;if\;v_{i\;}is\;placed\;on\;host\;h_{j}\\ 0,\;otherwise \end{array}\;\forall v_{i}\in V\;and\;\forall h_{j}\in H$$

(10) $$\sum_{i=1}^{n}x_{v_{i}h_{j}}=1,\;\forall h_{j}\in H$$

(11) $$\sum_{i=1}^{n}C_{v_{i}}^{d}\times x_{v_{i}h_{j}}\leq C_{h_{j}}^{d},\;\forall h_{j}\in H\;and\;\forall d\in D$$

(12) $$U_{t}^{d}\left(h_{j}\right)\leq Thr_{max}^{d}\left(h_{j}\right),\;\forall h_{j}\in H\;and\;\forall d\in D$$

where $\vartheta_{1}+\vartheta_{2}+\vartheta_{3}+\vartheta_{4}=1$ and $\vartheta_{1},\;\vartheta_{2},\;\vartheta_{3},\;\vartheta_{4}$ are the weight values. Constraint (10) ensures that the VM is allocated to only one host. Constraints (11) and (12) help ensure that each host meets the resource requirements of the VMs on it and does not exceed the maximum threshold $Thr_{max}^{d}\left(h_{j}\right)$.

## 4. Host Load Detection Based on Dynamic Load Mean

### 4.1. Dynamic Load Mean

Host load detection is a key step in VM consolidation. Any kind of resource overload (e.g., CPU, memory, network and storage) will greatly degrade the service performance and lead to SLAv. Therefore, the host load should be detected based on multi-dimensional resources. When hosts are overloaded, VM consolidation is performed to limit the resources utilization within a certain range to avoid a performance degradation caused by a resource overload. In addition, the host load dynamically changes and fluctuates with its hosted VMs workload. A short-term fluctuation will not affect the performance of the system; if each fluctuation triggers the VMs migration, it will make the VMs consolidation too aggressive and bring a negative impact that not only poses the risk of a host overload, but also affects the performance of the applications on the VM.

In order to solve the above problems, we propose a host load detection method based on the dynamic load mean (DLM-HLD). On the one hand, the DLM-HLD uses multi-dimensional resources to calculate the comprehensive load of the hosts. On the other hand, it considers the impact brought by the system load’s fluctuation, and uses the dynamic load mean within the recent sliding time window when calculating the resource’s load in each dimension. The sliding time window size can be dynamically adjusted according to the load fluctuation’s size, thus dynamically adjusting the load mean. The host $h_{j}$ comprehensive load $L\left(h_{j}\right)$ is calculated as shown in Equation (13).

(13) $$L\left(h_{j}\right)=\sum_{d\in D}\omega \left(d\right)\times U^{d}\left(h_{j},T\right)$$

(14) $$U^{d}\left(h_{j},T\right)=\frac{1}{T}\sum_{t\in T}U_{t}^{d}\left(h_{j}\right)$$

(15) $$T=\frac{\left|U^{d}\left(h_{j},T-1\right)-\max\left(Ur\right)\right|}{s}$$

where ω(d) is the weight coefficient of the resource d, and $U^{d}\left(h_{j},T\right)$ represents the resource d load mean in the sliding window T, which is calculated based on multiple discrete samples $Ur=\left\{U_{t_{1}}^{d}\left(h_{j}\right),U_{t_{2}}^{d}\left(h_{j}\right),\cdots ,U_{t_{i}}^{d}\left(h_{j}\right),\cdots ,U_{t_{k}}^{d}\left(h_{j}\right)\right\}$ in the sliding window time $T=\left\{t_{1},t_{2},\cdots ,t_{i},\cdots ,t_{k}\right\}$, where k=T/5 takes a positive value and samples are collected every 5 s. The sliding time window size T can be dynamically adjusted according to the absolute difference between the maximum value max(Ur) of the resource d in time T and the load mean $U^{d}\left(h_{j},T-1\right)$ in time T−1, as shown in Equation (15). The larger the absolute difference, the larger the sliding time window size, and the larger the samples number, then the better ability to withstand load fluctuations during the VM consolidation. The parameter s defines the sensitivity to changes in resource d. The smaller s is, the more sensitive it is to perceive changes.

Different resources have a different utilization on a host. The higher the resource utilization, the greater the impact on the host overload and the higher the weight is. The information entropy algorithm determines the weight according to the variation degree of the metric. The greater the variation degree, the greater the impact, the smaller the information entropy and the larger its weight. On the contrary, the smaller the variation degree, the smaller the impact, the larger the information entropy and the smaller its weight. In order to objectively perceive the comprehensive host load state, this paper uses information entropy to calculate the comprehensive host load $L\left(h_{j}\right)$, and the specific steps are as follows.

(1) The decision matrix is calculated as the following equation, where each row of the matrix records the load mean of the resource d corresponding to the host $h_{j}$, and each column of the matrix records the resource type.

(16) $$U=\left[\begin{array}{ccc} U^{d_{11}}\left(h_{j},T\right) & \cdots & U^{d_{1d}}\left(h_{j},T\right)\\ \vdots & \ddots & \vdots \\ U^{d_{k1}}\left(h_{j},T\right) & \cdots & U^{d_{kd}}\left(h_{j},T\right) \end{array}\right]$$

(2) The matrix is normalized to obtain the matrix, as shown below.

(17) $$R=\left[\begin{array}{ccc} u_{11} & \cdots & u_{1d}\\ \vdots & \ddots & \vdots \\ u_{k1} & \cdots & u_{kd} \end{array}\right]$$

where $u_{xy}=\frac{U^{d_{xy}}\left(h_{j},T\right)}{\sum_{x=1}^{k}U^{d_{xy}}\left(h_{j},T\right)}$.

(3) Calculate the y-th term entropy $E_{y}=-\frac{1}{lnk}\sum_{x=1}^{k}u_{xy}lnu_{xy}$, $E_{y}\in \left[0,1\right]$.
(4) The computational contribution degree is $d_{y}=1-E_{y}$, then the weight $\omega_{y}$ is shown in the following equation.

(18) $$\omega_{y}=\frac{d_{y}}{\sum_{y=1}^{d}d_{y}}$$

The $\omega_{y}$ is the weight of the resource d, and then the host $h_{j}$ comprehensive load $L\left(h_{j}\right)$ can be calculated by Equation (13).

### 4.2. Host Load Detection Base on Dynamic Load Mean

The pseudocode of the DLM-HLD is shown in Algorithm 1. First, we calculate the load mean $U^{d}\left(h_{j},T\right)$ of the host resource d; then, the host comprehensive load $L\left(h_{j}\right)$ is calculated based on $U^{d}\left(h_{j},T\right)$. Finally, based on the limit thresholds, all the hosts are divided into three categories: the overload, normal load and underload. Assume that the host comprehensive load upper and lower thresholds are $Thr_{max}$ and $Thr_{min}$, respectively, and the host resource d load upper and lower thresholds are $Thr_{d}^{max}$ and $Thr_{d}^{min}$, respectively. If $L\left(h_{j}\right)>Thr_{max}$ or $U^{d}\left(h_{j},T\right)>Thr_{d}^{max}$, the host is classified as overload set $H_{o}$; conversely, if $L\left(h_{j}\right)\leq Thr_{min}$ or $U^{d}\left(h_{j},T\right)\leq Thr_{d}^{min}$, the host is classified as underload set $H_{u}$. The remaining hosts are classified as the normal load set $H_{n}$.
**Algorithm 1** Host load detection algorithm DLM-HLD**Input:** host list H, upper threshold$\;Thr_{max}$ and $Thr_{d}^{max}$, lower threshold $Thr_{min}\;$and $Thr_{d}^{min}$ **Output:** overloaded hosts $H_{o}$, normal hosts $H_{n}$ and underloaded hosts $H_{u}$
Initialize: $H_{o}\leftarrow \emptyset$, $H_{n}\leftarrow \emptyset$, $H_{u}\leftarrow \emptyset$**for**$H_{i}$ **in** H **do** Base (15) compute T
 Base (14) compute $U^{d}\left(h_{j},T\right)$, t∈T and $U^{d}\left(h_{j},t\right)\in Ur$ Base (16), (17), (18) compute ω(d) Base (13) compute $L\left(h_{j}\right)$ **If** $L\left(h_{j}\right)>Thr_{max}$ or $U^{d}\left(h_{j},T\right)>Thr_{d}^{max}$ **then**    $H_{o}\leftarrow H_{i}$  **else if** $L\left(h_{j}\right)\leq Thr_{min}$ or $U^{d}\left(h_{j},T\right)\leq Thr_{d}^{min}$    $H_{u}\leftarrow H_{i}$  **else**    $H_{n}\leftarrow H_{i}$  **End if****End for****Return**$H_{o}$, $H_{u}$, $H_{n}$


## 5. The Proposed DLMM-VMC Algorithm

The following describes the main ideas of the DLMM-VMC. First, according to the DLM-HLD method, the hosts are divided into three categories: the overload, normal load and underload. Then, selecting the migration VMs and placement hosts based on the optimization ant colony algorithm. On the one hand, when selecting migration VMs, prioritize the VMs that make the greatest reduction in the overloaded resource utilization on the host, which effectively reduces the migration VMs number. In addition, to save energy, migrate as many VMs on underload hosts as possible to shut down more hosts. On the other hand, when selecting placement hosts, prioritize the hosts that make the best utilization of the resources on the host, which effectively reduces the resources waste. Finally, based on the multi-objective function proposed in Equation (8) and the optimized ant colony algorithm, the optimal solution is obtained. Define the mapping tuple set of migration VMs and placement hosts as $TC=\left\{\left(v_{m},h_{p}\right)\right\}$, where $v_{m}$ is a VM to be migrated and $h_{p}$ is a placement host for migration VMs to be placed. The elements in the collection TC are used as food for the ants. Ants search for solutions from TC and use objective function (8) to evaluate the solutions, and finally get the optimal solution. 

**Figure 1:**
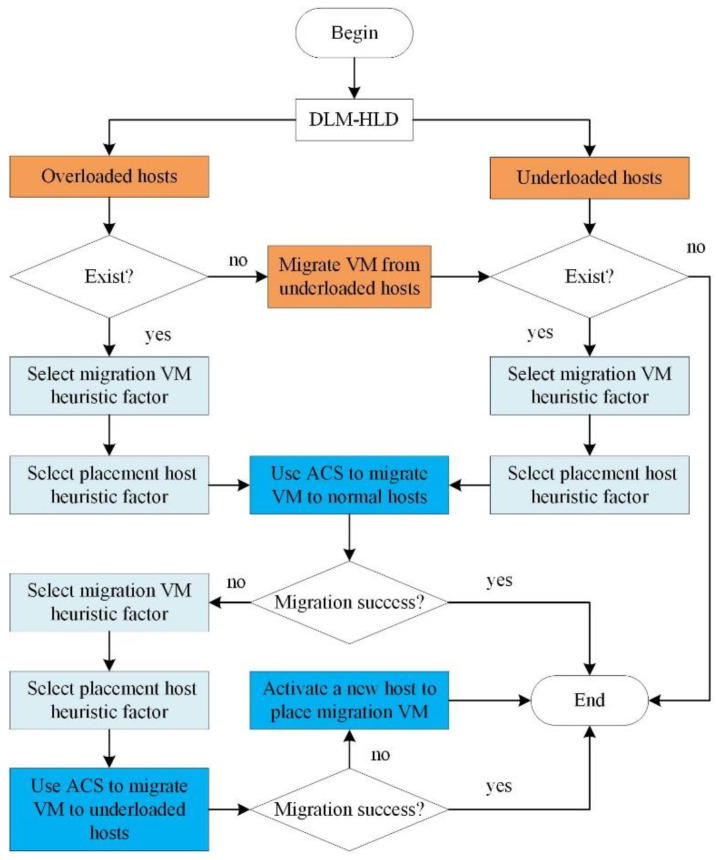
DLMM-VMC flow chart.

To reduce the time complexity of this method, we optimized the execution process of the ACS, as shown in Figure 1. This method restricts the solution search space of the ACS to a certain range of hosts instead of all the hosts based on the host load types output by the DLM-HLD algorithm. During the VM consolidation process, select the overloaded hosts and the underloaded hosts in turn, and when selecting the placement hosts for migration VMs, select the normal host, underloaded host and new host in turn. Therefore, compared with the original ant colony optimization algorithm, this method reduces the search range of the ants. In addition, to further optimize the execution efficiency for the VM consolidation problem, the heuristic factor takes into account both the migration VMs selection and placement hosts selection. On the one hand, the optimized heuristic factor selects a different migration VM selection heuristic factor based on the source host load type, which ensures minimizing the migration overhead when selecting the migration VMs. On the other hand, the optimized heuristic factor selects a different placement host selection heuristic factor based on the target host load type, which ensures a maximum resource utilization when selecting the placement hosts. The heuristic factor comprehensively considers the two key processes of the migration VMs selection and placement hosts selection, which helps to ensure the solution quality while reducing the blindness of the ant search to improve the execution efficiency of the ACS. In the following, detailed definitions of these factors are given.

### 5.1. Pheromone Definition

Pheromone $\tau_{ij}$ is the medium that ants communicate with each other. Ants find food sources by sensing other ants’ pheromones, and the higher the pheromone concentration, the greater the preference. Suppose that $\tau_{ij}$ denotes the pheromone on the combination $\left(v_{i},h_{j}\right)$ of VM $v_{i}$ and host $h_{j}$, the pheromone $\tau_{ij}$ value changes due to the new pheromone accumulation and the old pheromone volatilization. The local pheromone update rule is as follows.

(19) $$\tau_{ij}=\left(1-\rho \right)\cdot \tau_{ij}+\rho \cdot \tau_{0}$$

where ρ∈[0,1] is the pheromone volatility coefficient and $\tau_{0}$ is the initial pheromone that is a constant. Updating the local pheromone will reduce the pheromone concentration to avoid a premature convergence to suboptimal solutions.

After all the ants have constructed their solutions, the global optimal solution is obtained according to the objective function and the global pheromone is updated using the global optimal solution to enhance the experience of the global optimal solution. The global pheromone update rule is as follows.

(20) $$\tau_{ij}=\left(1-\rho \right)\cdot \tau_{ij}+\rho \cdot F\left(X^{+}\right)$$

where $X^{+}$ is the global optimal solution.

### 5.2. Definition of Heuristic Factor

In addition to the pheromone, the heuristic factor $\eta_{ij}$ is another very critical factor in an ant colony algorithm. The heuristic factor represents the expectation that the VM $v_{i}$ is assigned to host $h_{j}$. The larger the heuristic information is, the greater the corresponding behavior probability is. Therefore, a reasonable setting of the heuristic factor can reduce the search blindness and improve the search efficiency of the ant colony. This paper comprehensively considers the service performance and energy consumption, and sets the heuristic factor as shown in the following equation, which consists of two parts: the selection of migration VMs and placement hosts.

(21) $$\eta_{ij}=\lambda \cdot \eta_{v}\left(h_{i},-v_{i}\right)+\left(1-\lambda \right)\cdot \eta_{h}\left(h_{j},+v_{i}\right),\lambda \in \left[0,1\right]$$

where $\eta_{v}\left(h_{i},-v_{i}\right)$ is the migration VMs selection heuristic factor, which indicates that the VM $v_{i}$ is migrated from the host $h_{i}$, and $\eta_{h}\left(h_{j},+v_{i}\right)$ is the placement hosts selection heuristic factor, which indicates that the VM $v_{i}$ is migrated to the host $h_{j}$. λ is the relative weight to measure the relative importance of the two. The settings of $\eta_{v}\left(h_{i},-v_{i}\right)$ and $\eta_{h}\left(h_{j},+v_{i}\right)$ are described in detail below.

#### 5.2.1. Migration VM Selection

For overloaded hosts, any kind of resource overload may affect the QoS and result in SLAv. In addition, an improper policy for selecting migration VMs will cause too many VMs to be migrated, which increases the migration overhead. Therefore, for overloaded hosts, the main strategy for selecting a migration VM is to minimize the VM migration number and time under the premise of comprehensively considering multi-dimensional resources, and quickly restores the host from the overload state to the normal state. Therefore, we define the migration VM selection heuristic factor for the overload hosts as follows.

(22) $$\eta_{v}\left(h_{i},-v_{i}\right)=\sum_{d\in D}\omega \left(d\right)\times \left(U_{o}^{d}\left(h_{i}\right)-U_{o}^{d}\left(h_{i},-v_{i}\right)\right)-T_{mig}\left(h_{i},-v_{i}\right)$$

where $U_{o}^{d}\left(h_{i}\right)$ is the utilization of the resource d of the overloaded host $h_{i}$, $U_{o}^{d}\left(h_{i},-v_{i}\right)$ is the utilization of the resource d after the host $h_{i}$ migrating out of the VM $v_{i}$, $\sum_{d\in D}\omega \left(d\right)\times \left(U_{o}^{d}\left(h_{i}\right)-U_{o}^{d}\left(h_{i},-v_{i}\right)\right)$ is the load’s comprehensive descending gradient of the host $h_{i}$ after the VM $v_{i}$ is migrated out from the host $h_{i}$ and ω(d) is the weight value obtained based on Equation (18); the greater the descent gradient, the greater the probability of the VM $v_{i}$ being selected. Additionally, considering the migration time $T_{mig}\left(h_{i},-v_{i}\right)$ as a migration VM selection factor, this paper uses the migration time evaluation model proposed in the literature [41] to calculate $T_{mig}\left(h_{i},-v_{i}\right)$, which evaluates the VM migration time based on the current memory usage, dirty page and data transfer rate. $\eta_{v}\left(h_{i},-v_{i}\right)$ comprehensively considers the VM migration number and time. When selecting a migration VM, the faster the host overload state decreases and the shorter the migration time, the more likely the VM will be selected.

For underload hosts, in order to minimize the underload host number, preference is given to VMs that can significantly reduce the host’s resources utilization after migration to shut down the host. Therefore, we define the migration VM selection heuristic factor on the underload hosts as follows.

(23) $$\eta_{v}\left(h_{i},-v_{i}\right)=\sum_{d\in D}\left(1-U_{u}^{d}\left(h_{i},-v_{i}\right)\right)$$

#### 5.2.2. Placement Host Selection

When any resource usage of a host is overloaded, the host performance will drop rapidly. Therefore, when the normal load host is selected as the placement host, the one with more remaining resources is preferred. Additionally, consider resources waste and choose the one with the less resources waste. We comprehensively consider the QoS and resources waste and set the heuristic factor for normal load hosts in the following formula.

(24) $$\eta_{h}\left(h_{j},+v_{i}\right)=\left|1-L\left(h_{j},+v_{i}\right)-W\left(h_{j}\right)\right|$$

where $1-L\left(h_{j},+v_{i}\right)$ and $W\left(h_{j}\right)$ are the remaining comprehensive load and resources waste of host $h_{j}$ after deploying VM $v_{i}$. The larger the $\eta_{h}\left(h_{j},+v_{i}\right)$, the greater the remaining comprehensive load, and the less resources waste there is.

For the underload hosts, their resources utilization are low and resources competition are weak, which can guarantee a QoS but cause a waste of resources and energy. Therefore, when selecting underload hosts as the placement hosts, the hosts with a higher resource utilization after deploying VMs are preferred to fully utilize the resources. The corresponding heuristic factor is defined as follows.

(25) $$\eta_{h}\left(h_{j},+v_{i}\right)=\left|L\left(h_{j},+v_{i}\right)-W\left(h_{j}\right)\right|$$

### 5.3. Pseudo-Random Proportion Rule

According to the heuristic factor and pheromone information, the ants construct the solution according to the following pseudo-random proportion rule.

(26) $$\left(v_{m},h_{p}\right)=\{\begin{array}{c} arg\;max\left\{\tau_{ij}^{\alpha }\cdot \eta_{ij}^{\beta }\right\},if\;q\leq q0\\ \left(v_{i},h_{j}\right),\;ohterwise \end{array}$$

where q∈[0,1] is a uniformly distributed random number and q0∈[0,1] is a fixed parameter determining the relative importance of cumulative experience and random selection. The α and β indicate the importance of the pheromone and heuristic factor. When q≤q0 is called an exploitation, it is helping the ants to converge quickly to a high-quality solution, otherwise it is called an exploration, in which ants randomly select a tuple $\left(v_{i},h_{j}\right)$ according to the probability distribution defined in the following equation, helping the ants to discover more new choices.

(27) $$p_{mp}^{k}=\{\begin{array}{c} \frac{\tau_{ij}^{\alpha }\cdot \eta_{ij}^{\beta }}{\sum_{\left(v_{i},h_{j}\right)\in TC_{allow}^{k}}\left(\tau_{ij}^{\alpha }\cdot \eta_{ij}^{\beta }\right)},\;if\;\left(v_{i},h_{j}\right)\in TC_{allow}^{k}\\ 0,\;ohterwise \end{array}$$

where $TC_{allow}^{k}$ denotes the set of tuples that ant $ant_{k}$ is allowed to traverse, and $p_{mp}^{k}$ denotes the probability that ant $ant_{k}$ selects the tuple $\left(v_{m},h_{p}\right)$ next.

### 5.4. VM Consolidation Algorithm

The pseudo-code of the DLMM-VMC is shown in Algorithm 2. The input host sets are obtained by Algorithm 1. The algorithm initializes the iterations number nI, ants number nA and $\tau_{0}$ (line 1). In each iteration, nA ants traverse the overloaded and underloaded hosts in turn, calculate heuristic factors based on the source and target host load types and reconstruct new mapping relationships between the VMs and hosts (lines 3–38). First, the VM consolidation is performed for the overloaded hosts (lines 4–20), and heuristic factors are calculated according to the overload host type (lines 7 and 11). Then, a VM consolidation is performed for the underload hosts (lines 21–36), and the heuristic factors are calculated according to the underload host type (lines 23 and 28). When selecting placement hosts, this algorithm selects the normal host and the underload host in turn (lines 5, 10, 22 and 27). After each ant constructs the solution, the local pheromone is updated (lines 19 and 35), and when all the ants have constructed the solutions, all ant-specific solutions are added to the solution set M (line 37). The solution set in M is evaluated using the objective function to obtain the global optimal solution $X^{+}$ (line 39), and then the global pheromone (line 40) is updated using $X^{+}$. When all the iterations are executed, $X^{+}$ is the final optimal solution.
**Algorithm 2** VM consolidation algorithm DLMM-VMC**Input:** overloaded host set $H_{o}$, normal host set $H_{n}$ and underloaded host set $H_{u}$. **Output:** $X^{+}$Initialize: nI, nA, $\tau_{0}$, $X^{+}\leftarrow \emptyset$, $X^{k}\leftarrow \emptyset$ and M←∅.**for** i=1 to nI **do** **for** j=1 to nA **do**  **while** $H_{o\;}!=\emptyset$ **do**   $TC\leftarrow \{\left(v_{m},h_{p}\right)|\forall v_{m}\in VM\left(h_{j}\right),h_{j}\in H_{o}\;and\;\forall h_{p}\in H_{n}\}$   Based on Equations (21), (22) and (24), compute heuristic $\eta_{mp}$ and $\forall \left(v_{m},h_{p}\right)\in TC$.   Based on Equation (27), compute $p_{mp}$ and $\forall \left(v_{m},h_{p}\right)\in TC$.   Based on Equation (26), select $\left(v_{m},h_{p}\right)\in TC$.   **If** $\left(v_{m},h_{p}\right)\;is\;null,$ **then**$TC\leftarrow \{\left(v_{m},h_{p}\right)|\forall v_{m}\in VM\left(h_{j}\right),h_{j}\in H_{o}\;and\;\forall h_{p}\in H_{u}\}$.    Based on Equations (21), (22) and (25), compute heuristic $\eta_{mp}$, $\forall \left(v_{m},h_{p}\right)\in TC$.    Based on Equation (27), compute $p_{mp}$ and $\forall \left(v_{m},h_{p}\right)\in TC$.    Based on Equation (26), select $\left(v_{m},h_{p}\right)\in TC$.    **If** $\left(v_{m},h_{p}\right)\;is\;null,$ **then**      Break    **End if**   **End if**   Update mapping relation matrix X.   Update local pheromone using (19).  **End while**  **while** $H_{u}\;!=\emptyset$ **do**   $TC\leftarrow \{\left(v_{m},h_{p}\right)|\forall v_{m}\in VM\left(h_{j}\right),h_{j}\in H_{u}\;and\;\forall h_{p}\in H_{n}\}$   Based on Equations (21), (23) and (24), compute heuristic $\eta_{mp}$ and $\forall \left(v_{m},h_{p}\right)\in TC$.   Based on Equation (27), compute $p_{mp}$ and $\forall \left(v_{m},h_{p}\right)\in TC$.   Based on Equation (26), select $\left(v_{m},h_{p}\right)\in TC$.   **If** $\left(v_{m},h_{p}\right)\;is\;null,$ **then**    $TC\leftarrow \{\left(v_{m},h_{p}\right)|\forall v_{m}\in VM\left(h_{j}\right),h_{j}\in H_{u}\;and\;\forall h_{p}\in H_{u}\},\;j\neq d$    Based on Equations (21), (23) and (25), compute heuristic $\eta_{mp}$ and $\forall \left(v_{m},h_{p}\right)\in TC$.    Based on Equation (26), select $\left(v_{m},h_{p}\right)\in TC$.    **If** $\left(v_{m},h_{p}\right)\;is\;null,$ **then**      Break    **End if**   **End if**   Update mapping relation matrix X.   Update local pheromone using (19).  **End while**  $M\leftarrow M\cup^{\;}\left\{X^{k}\right\}$ **End for** $X^{+}\leftarrow \arg max_{X^{k}\in M}\left\{F\left(X^{k}\right)\right\}$. Based on Equation (20), update global pheromone using $X^{+}$.**End for**


As shown in Algorithm 2, we can conclude that the maximum time complexity of this algorithm is O(nI·nA·m·n). Where nI is the number of iterations, nA is the number of ants, m is the number of VMs and n is the number of hosts. In line 4 and line 21, the while loop traverses the overloaded and underloaded hosts with the number of traversals less than n, and then in line 5, line 10, line 22 and line 27, the VMs on the overloaded and underloaded hosts are traversed in turn to construct the solution space with the number of traversals less than m·n. Because we handle overloaded hosts and underloaded hosts separately, select normal load hosts and underloaded hosts sequentially when selecting the placement hosts. Therefore, the number of VMs and hosts traversed each time is less than m and n, respectively, and the final complexity of the DLMM-VMC is less than or equal to O(nI·nA·m·n).

## 6. Performance Analysis and Discussion

### 6.1. Experimental Setup

The proposed algorithm was evaluated using the simulator CloudSim [30], which is a cloud computing environment simulation framework that can simulate most of the resources and behaviors of the cloud systems.

This experiment simulated a cloud data center with two types of hosts. The host types are HP ProLiant G4 and ProLiant G5, and the details of their configurations are shown in Table 1 and the energy consumption characteristics are shown in Table 2. The hosts were connected through a gigabit network. Four types of Amazon EC2 VMs [17] were used, and their configuration information is shown in Table 3. After the VM instances were created, they were initially deployed based on the resource requirements of the VM type.

To verify the validity of the proposed algorithm, the real-world workload dataset of the Google cluster data (GCD) [42] was used in the experiment. The GCD provided real tracking data for approximately a month in May 2011, which was tracked every five minutes and tracked multiple resources utilization, such as the CPU and memory. The data of different days were randomly selected from the processed data. The statistical characteristics of the 1600 VMs critical resources are shown in Table 4. The algorithm related parameter settings are shown in Table 5. Here, we set ϑ_i = 0.25 and d = 2, which indicates that the multiple objectives have the same weight and consider using the CPU and memory resources.

### 6.2. Performance Metrics

The service level agreement (SLA) refers to an agreement reached between the cloud service providers and users on services, priorities and responsibilities. If the SLA is violated, the users’ interests cannot be guaranteed, and the cloud service providers may pay expensive fines to users as compensation. Therefore, the SLA is an important metric to measure a data center’s QoS. SLAv [17] are an independent metric to measure SLA violations, which is measured from two aspects: the SLA violation time caused by the host overload (SLAHv) and the performance degradation caused by the VM migration (SLAMv). These two aspects are independent and have the same impact on SLAv. Therefore, the total SLAv are calculated as follows:

(28) SLAv=SLAHv×SLAMv

The SLAHv indicates the percentage of time when the CPU or memory usage of a host reached 100%; meanwhile, the SLAMv indicate the overall performance degradation caused by the VM migration. The SLAHv and SLAMv values are calculated as follows:

(29) $$SLAHv=\frac{1}{n}\overset{n}{\underset{j=1}{\sum }}\frac{T_{h_{j}\;}}{T_{a_{j}}}$$

(30) $$SLAMv=\frac{1}{m}\overset{m}{\underset{i=1}{\sum }}\frac{C_{v_{i}}}{C_{a_{i}}}$$

where n and m indicate the numbers of hosts and VMs in a data center, respectively; $T_{h_{j}}\;$and $T_{a_{j}}$ represent the number of time when the host utilization reached 100% and the total running time, respectively; $C_{v_{i}}$ represents the capacity of the unfulfilled resource requests caused by the VM migration, which is the estimation of the performance degradation caused by the VM migration; and $C_{a_{i}}$ is the total CPU requirement for a VM $v_{i}$ during its lifetime. Studies have shown that [17] SLAMv can be set to 10% of the CPU utilization during the VM migration. 

The energy consumption in the VM consolidation is an important evaluation metric. However, when optimizing the energy consumption, SLAv need to be balanced. The comprehensive evaluation metric PSV is calculated from the combination of the total energy consumption and SLAv, and is defined as follows.

(31) PSV=P×SLAv

where *P* is the total energy consumption according to Equation (3); when the *PSV* value is low, it indicates that the data center has a good performance in terms of the energy consumption and QoS.

In addition, the network communication overhead and migration overhead are also the optimization objectives in this paper, which are evaluated based on Equations (6) and (7), respectively. The network communication overhead $a\left(v_{i},v_{j}\right)$ between VMs $v_{i}$ and $v_{j}$ is calculated by referring to the literature [43].

### 6.3. Performance of DLM-HLD

**Figure 2:**
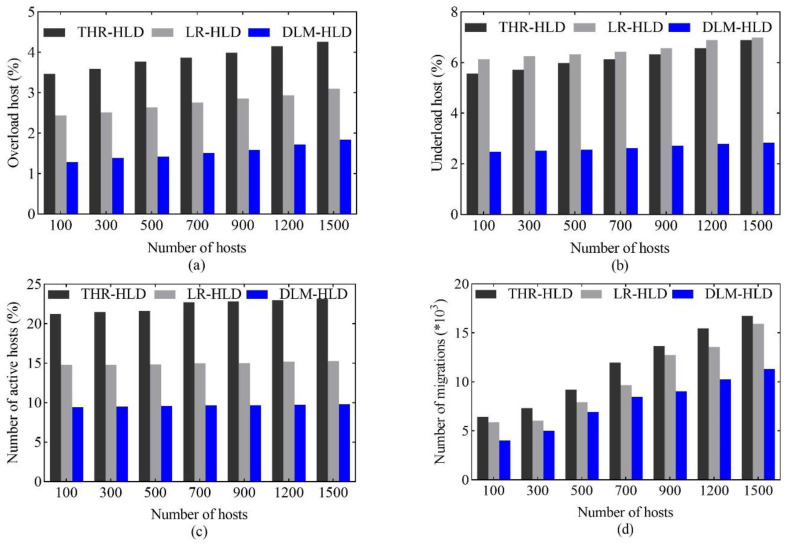
The number of (**a**) overloaded hosts, (**b**) underloaded hosts, (**c**) active hosts and (**d**) migrations number.

In this experiment, data centers of different sizes were used to evaluate the performance of the DLM-HLD algorithm. The number of hosts in a data center varied from 100 to 1500, and each host was initially deployed with two VMs on average. The experiment focuses on the impact of the dynamic load mean on the overloaded, underloaded, active hosts number and the migration number in different sizes of data centers. The DLM-HLD scheme was compared with the static and dynamic threshold detection methods proposed in [17]. The static threshold detection method (THR-HLD) set the maximum utilization of the CPU and memory to 80%. The dynamic threshold detection method (LR-HLD) estimated the threshold using the local regression (LR) method and detected overloaded hosts according to the estimated CPU and memory utilization values. The VM consolidation test was performed every 5 min, and the test results were recorded over 24 h. Figure 2 shows the test results.

According to Figure 2a,b, compared with the THR-HLD and LR-HLD algorithms, the DLM-HLD algorithm proposed in this paper detected the least number of overloaded or underloaded hosts in the VM consolidation. For a 1500-node data center, the DLM-HLD detected 56.8% and 40.6% fewer overloaded hosts and 58.9% and 59.5% fewer underloaded hosts compared to the THR-HLD and LR-HLD, respectively. The THR-HLD algorithm is based on the current resources utilization as the criterion for detecting a host overload or underload, without considering the dynamic changes in the resources load. As long as the current resource utilization exceeds the set threshold, the host is judged as an overload or underload, and even occasional load fluctuations will detect the hosts overload or underload, which leads to the misjudgment of the hosts overload or underload in the VM consolidation, thus increasing the number of overloaded or underload hosts. Although the LR-HLD can predict a future resources utilization, it cannot predict occasional load fluctuations. The DLM-HLD algorithm considers the dynamic load mean of resources over a period, which not only accurately judges the trend of resources usage but also filters out occasional load fluctuations, thus effectively reducing the misjudgment in the host load detection.

Next, the impact of the dynamic load mean (DLM) on the number of active hosts in different sizes of data centers was analyzed. As shown in Figure 2c, the number of hosts that need to be activated using the DLM-HLD method was the smallest. From the above analysis, we know that the THR-HLD and LR-HLD algorithms detected more overloaded and underloaded hosts. However, each VM consolidation required a VMs migration on the overloaded and underloaded hosts, which led to the migration of more VMs, of which Figure 2d shows the result, and finally more hosts were activated when more migration VMs were placed.

### 6.4. Multi-Objective Optimization Performance

**Figure 3:**
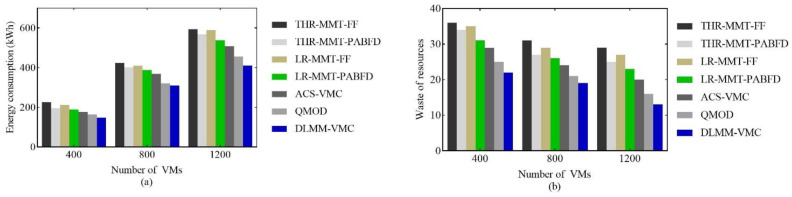
The comparison of (**a**) energy consumption and (**b**) resources waste.

This section evaluated the multi-objective optimization performance of the DLMM-VMC. Four heuristic algorithms and two meta-heuristic algorithms were used as the comparison benchmarks. For heuristic algorithms, host load detection used two algorithms: the static threshold THR and dynamic threshold LR [17]. The migration VMs selection used the minimum migration time algorithm MMT [17], and the placement host selection used both the FF [3] and PABFD [17] algorithms. The two meta-heuristic algorithms were ACS-VMC [40] and QMOD [13], respectively. The data center was sized to deploy 400, 800 and 1200 VMs based on 400 physical servers for testing. The VM consolidation experiments were executed every 5 min, and the test results were recorded over 24 h, as shown in Figure 3.

Figure 3a shows the energy consumption comparison. For the data center with 1200 VMs, the DLMM-VMC reduced the energy consumption by 30.8%, 27.7%, 30.3%, 19.1%, 23.5%, 19.3% and 9.8% compared with the THR-MMT-FF, THR-MMT-PABFD, LR-MMT-PABFD, ACS-VMC and QMOD, respectively. On the one hand, energy consumption was a major optimization objective in the DLMM-VMC, and the DLM-DLH method effectively reduced the number of active hosts. In addition, the resources waste was also our optimization objective. Figure 3b shows that the DLMM-VMC method has the least resources waste, which proves that the DLMM-VMC makes full use of resources so that it can minimize the active hosts’ number when deploying the same number of VMs. On the other hand, the DLMM-VMC optimized the heuristic factor of the ant colony algorithm. When selecting the placement hosts, the optimization heuristic factor fully considered the comprehensive resources utilization and resources waste of the host and selected the host with less resources waste under constraints. Therefore, compared with other algorithms, the DLMM-VMC effectively reduced the energy consumption.

**Figure 4:**
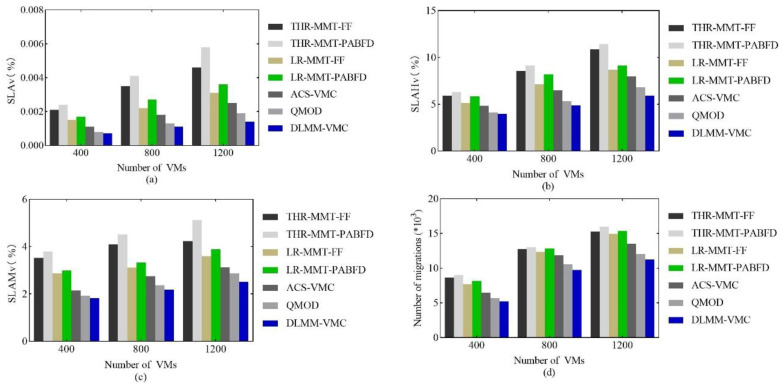
The comparison of (**a**) SLAv, (**b**) SLAHv, (**c**) SLAMv and (**d**) the migrations number.

Figure 4a illustrates the SLAv comparison. The results show that the DLMM-VMC has the best performance in SLAv, followed by the QMOD, and the THR-MMT-PABFD has the worst performance. For the data center with 1200 VMs, the DLMM-VMC SLAv were 73.6% of the QMOD, but was only 24.1% of the LR-MMT-PABFD, which proved that the DLMM-VMC algorithm effectively guaranteed the QoS. The SLAv were composed of SLAHv and SLAMv. In order to analyze the SLAv in more detail, we further analyzed the SLAHv and SLAMv.

Figure 4b illustrates the SLAHv comparison. These results show that DLMM-VMC algorithm has the lowest SLAHv, which indicates that the DLMM-VMC has a significant improvement in ensuring the host’s QoS. Because the DLMM-VMC considered the multi-dimensional resources of the host in the host overload detection, which avoided the SLAv caused by any kind of resource overload on the host, it effectively guaranteed the host’s QoS. Figure 4c shows that the DLMM-VMC has the best performance in SLAMv compared to the other algorithms, which indicates that the DLMM-VMC effectively reduces the impact of migration on the VMs QoS. On the one hand, the objective function defined by the DLMM-VMC tends to minimize the VM migrations number; Figure 4d demonstrated the result. On the other hand, the DLM-DLH effectively avoided unnecessary VM migrations caused by the load fluctuation. In addition, the DLMM-VMC ensured that the overloaded hosts were quickly and accurately restored to a normal load level with a minimal migration overhead based on the optimized heuristic factors when selecting migration VMs. Therefore, compared with other comparison algorithms, the DLMM-VMC had obvious advantages in SLAv.

**Figure 5:**
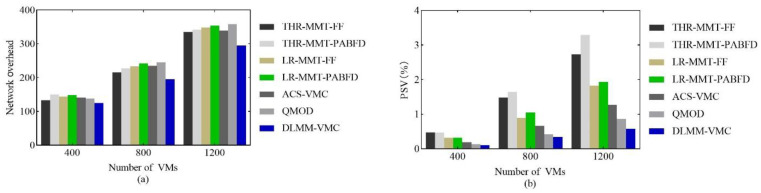
The comparison of (**a**) network overhead and (**b**) PSV.

Figure 5a shows the network overhead comparison. Based on the tree network topology, the results show that the DLMM-VMC has the minimum network overhead, which proves that the network overhead model proposed in this paper effectively reduces the network communication cost. The DLMM-VMC placed the interdependent VMs close to each other so as to reduce the number of network elements that pass through during the network’s communication. It is well known that transmission information is processed and forwarded as it passes through the network’s elements, which increases the corresponding transmission delay. If VMs that communicate with each other are maximally deployed on the same server, the communication traffic handled by the network elements in the data center is greatly reduced, which not only reduces the overhead of the network resources but also improves the overall communication performance of the data center.

Figure 5b shows the comprehensive performance comparison. The results show that the DLMM-VMC has the lowest value, indicating that its comprehensive performance is the best. The PSV was composed of the total energy consumption P and SLAv. The above analysis shows that P and SLAv achieve the optimal results compared to other algorithms. Therefore, the PSV is also optimal.

### 6.5. Execution Efficiency Analysis

**Figure 6:**
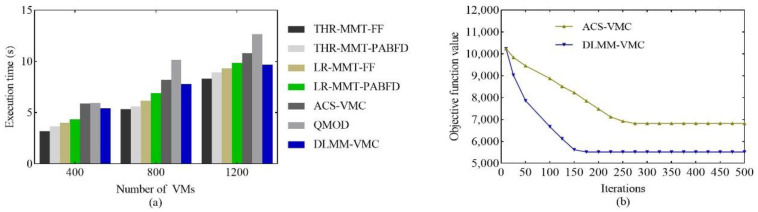
The comparison of (**a**) execution time and (**b**) convergence performance.

In order to deeply analyze the efficiency of the DLMM-VMC, the execution time was analyzed, as shown in Figure 6a. Due to the low time complexity, the four heuristic algorithms were shorter than the three meta-heuristic algorithms. However, the DLMM-VMC was better than the other two meta-heuristics and was close to the three heuristics. The DLMM-VMC algorithm limited the solution search space of the ant colony based on the host load types, which effectively improved the execution efficiency.

In addition, we compared the DLMM-VMC with the ACS-VMC in terms of the convergence. We calculated the objective function value according to Equation (8) and run the two algorithms 10 times separately. The number of VMs was set to 400. As seen in Figure 6b, both the DLMM-VMC and ACS-VMC converged in 500 iterations, and the DLMM-VMC solution was smaller. The DLMM-VMC algorithm converges significantly faster than the ACS-VMC algorithm, which starts to converge after 150 iterations, and the ACS-VMC algorithm has a convergence trend after 260 iterations. It can be seen that the DLMM-VMC has been improved in terms of the algorithm convergence performance.

## 7. Conclusions

This paper focuses on how to optimize the energy consumption, resource utilization, QoS, migration overhead and network communication overhead in cloud data centers, and thus proposes a DLMM-VMC algorithm to do so. The DLMM-VMC constructs the VM consolidation problem as a multiple-objective optimization problem. Fist, a host load detection method based on the dynamic load mean is proposed to objectively and accurately evaluate the real load state of the hosts, which avoids the deficiency of only considering single-dimensional resources in VM consolidation and also optimizes the problem of unnecessary VM migrations caused by system load fluctuations. Then, the optimized ant colony algorithm is proposed to obtain the optimal mapping scheme between the hosts and the VMs. In this process, the heuristic factor and the execution process of the ACS are optimized to achieve the improvement in the multiple objective optimization and execution efficiency. Finally, the experimental results show that the DLMM-VMC is effective in reducing the energy consumption, optimizing resources utilization, guaranteeing a QoS and reducing a migration overhead and network communication overhead compared with other algorithms.

This paper ignores the energy consumption generated by other devices in the data center and the impact on the system’s performance, such as the network elements and refrigeration equipment. In the future, we will comprehensively consider various factors to conduct VM consolidation research to further optimize the energy consumption.

## Figures and Tables

**Table 1:** Host Configuration Information.

Types	CPU Type	Frequency (GHz)	Core	RAM(GB)
HP ProLiant G4	Inter Xeon 3040	1.86	2	4
HP ProLiant G5	Inter Xeon 3075	2.66	2	4

**Table 2:** Energy consumption of experimental host (watt).

Types	0%	10%	20%	30%	40%	50%	60%	70%	80%	90%	100%
HP G4	86	89.4	92.6	96	99.5	102	106	108	112	114	117
HP G5	93.7	97	101	105	110	116	121	125	129	133	135

**Table 3:** VM configuration information.

Types	CPU Frequency (MIPS)	RAM(GB)
High-CPU medium instance	2500	0.85
Extra large instance	2000	3.75
Small instance	1000	1.7
Micro instance	500	0.613

**Table 4:** GCD data set statistical features.

Resource	Number of VMs	Mean (%)	St. Dev. (%)	Median (%)
CPU	1600	21.23	12.78	18
Memory	1600	18.57	15.83	13

**Table 5:** Parameters of proposed algorithm.

$\vartheta_{i}$	λ	s	**α**	**β**	nI	nA
0.25	0.5	1	1	1.5	10	10

## Data Availability

Data will be available upon request through correspondence email.
